# Supplementary material for: The Mouse CircGHR Regulates Proliferation, Differentiation and Apoptosis of Hepatocytes and Myoblasts
Source: Genes (Basel). 2023 May 31;14(6):1207. doi: 10.3390/genes14061207 (PMC10298241; doi:10.3390/genes14061207)
Supplement: Supplementary file 1 [file genes-14-01207-s001.zip › genes-2338546-supplementary.pdf]

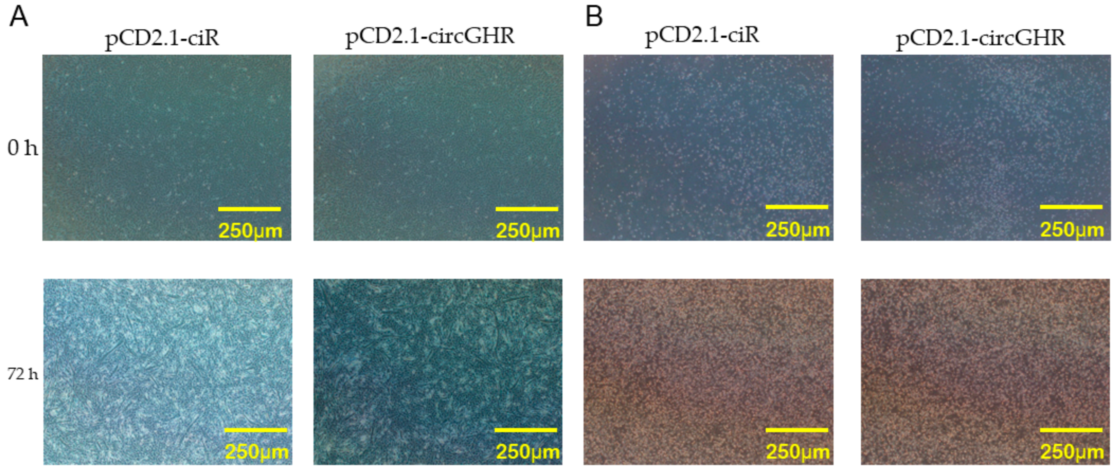

Sumpplement Figure S1. Images of mouse C2C12 and NCTC1469 cell lines (A) Images of mouse C2C12 cell lines at 0 h and 72 h after transfection with circGHR;(B) Images of mouse NCTC1469 cell lines at 0 h and 72 h after transfection with circGHR

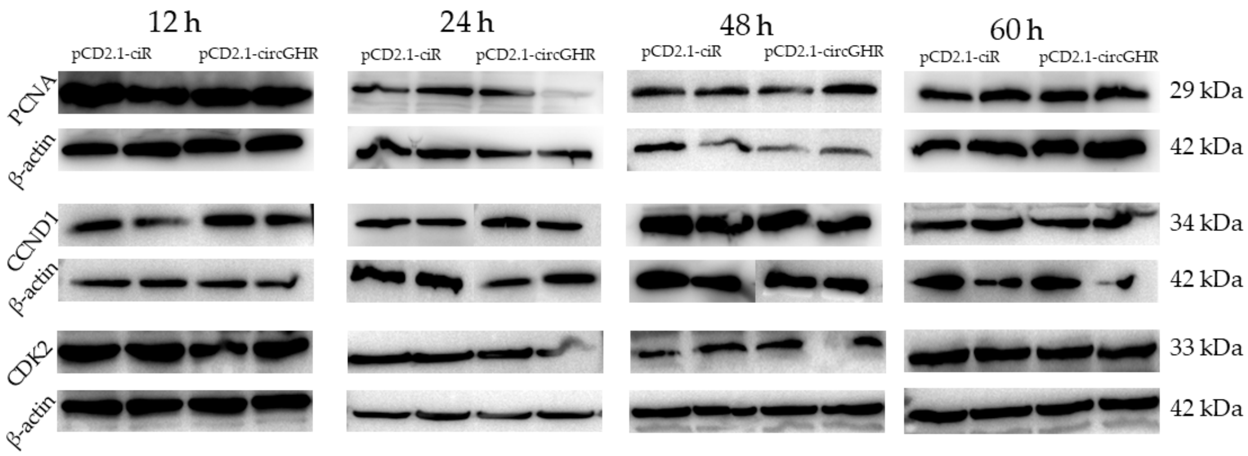

Sumpplement Figure S2. Protein expression levels of CCND1 detected by western blot at different time point after transfecting pCD2.1-ciR and pCD2.1-circGHR in mouse NTCT1469 cells.;

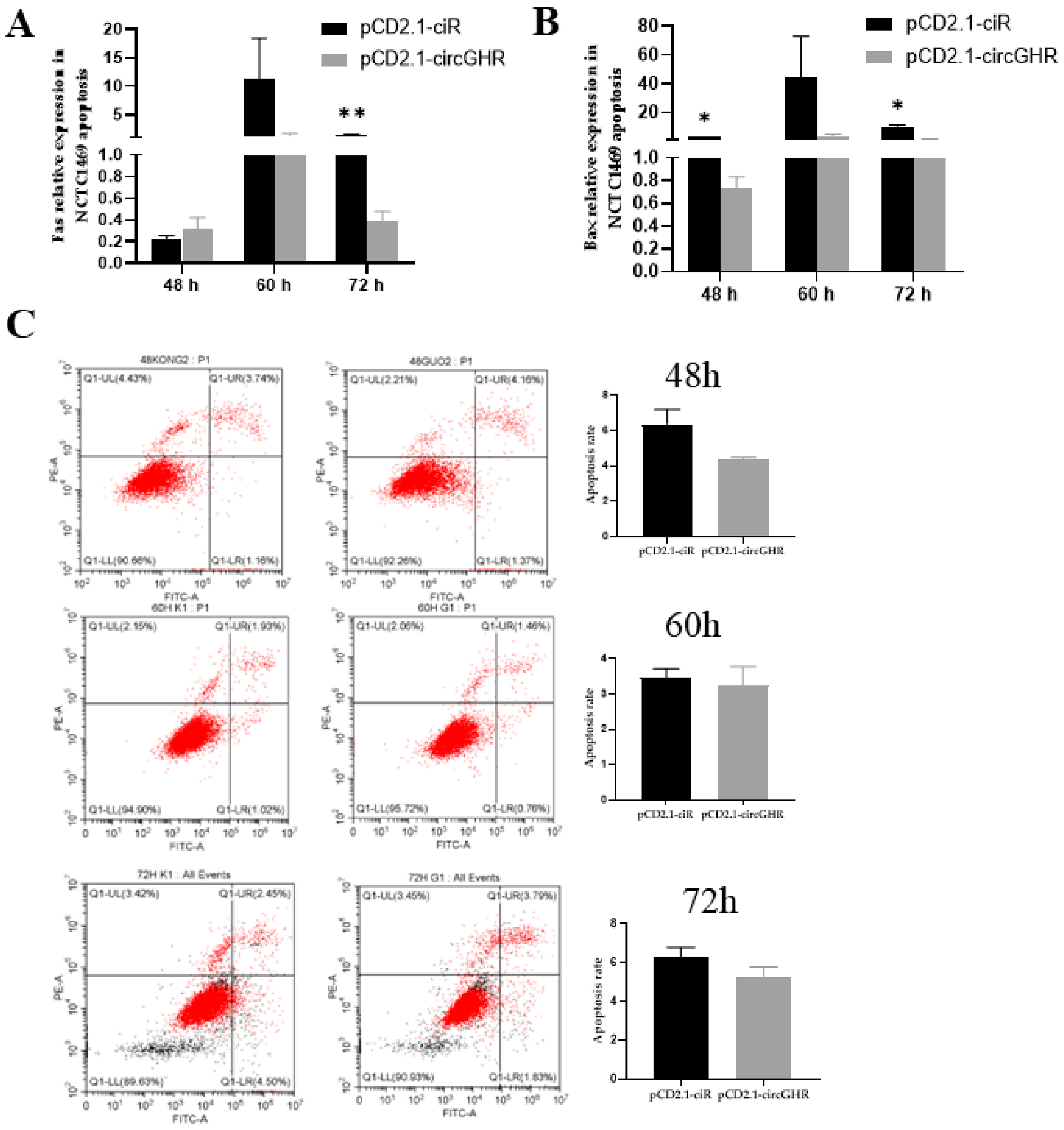

Sumpplement Figure S3. The effect of circGHR on apoptosis of NCTC1469 cells.

(A) The relative expression level of *Fas* gene at 48, 60 and 72 hours after overexpression of circGHR; (B) The relative expression level of *Bax* gene at 48, 60 and 72 hours after overexpression of circGHR; (C) The apoptosis rate of cells in empty vector group and overexpression vector group at 48,60 and 72 hours. n = 4. The qRT-PCR results were calculated using the $2^{-\Delta\Delta CT}$ method. **: $P < 0.01$, *: $P < 0.05$.

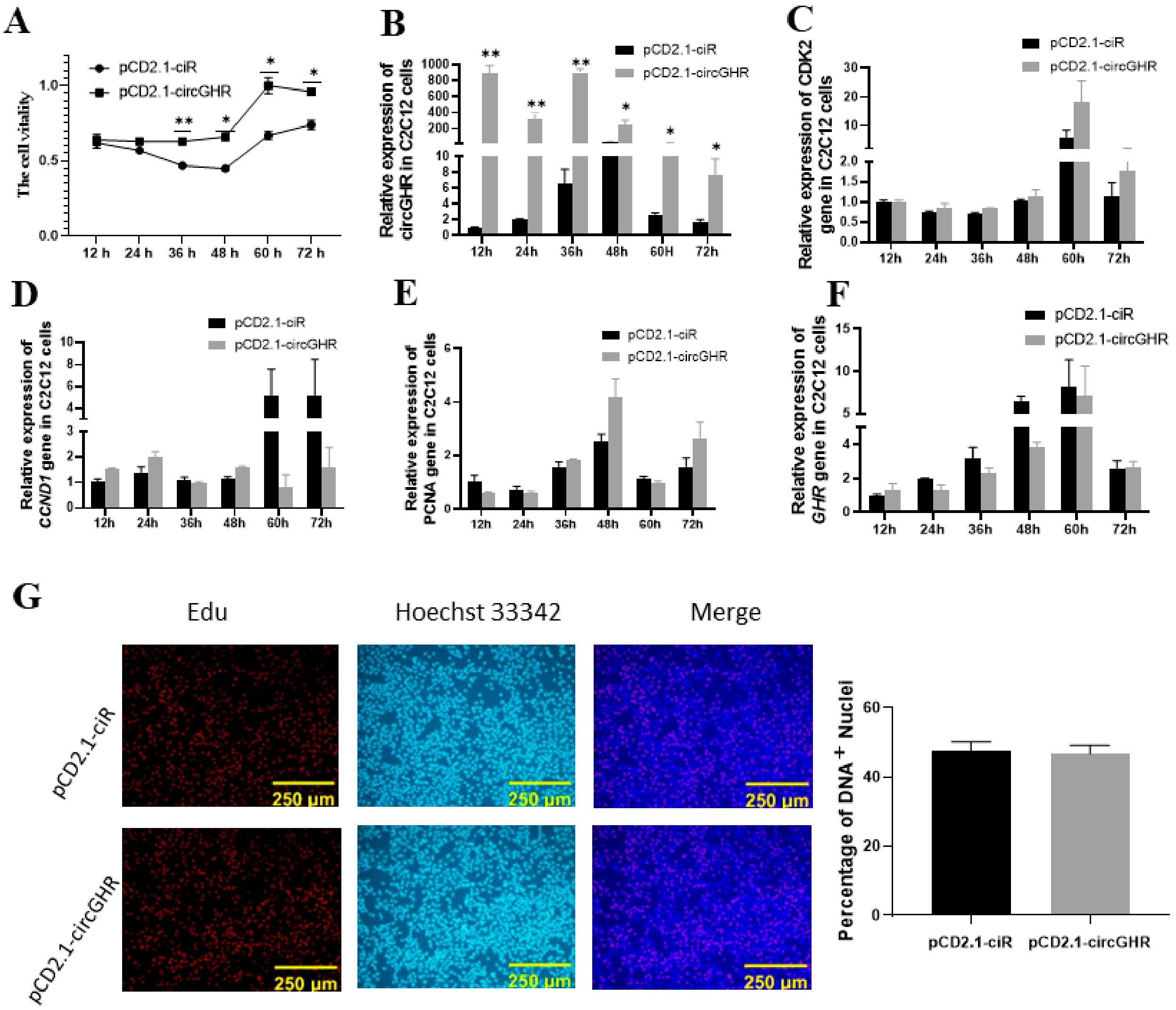

Sumpplement Figure S4. Effect of the mouse circGHR on the proliferation of C2C12 cells.

(A) CCK-8 test results after transfection of pCD2.1-ciR and pCD2.1-circGHR; (B) circGHR expression changes after transfection of pCD2.1-ciR and pCD2.1-circGHR; (C) The expression level of *CDK2* after transfection of pCD2.1-ciR and pCD2.1-circGHR; (D) The expression level of *CCND1* after transfection of pCD2.1-ciR and pCD2.1-circGHR; (E) The expression level of *PCNA* after transfection of pCD2.1-ciR and pCD2.1-circGHR; (F) *GHR* mRNA gene expression changes after pCD2.1-ciR and pCD2.1-circGHR transfection; (G)Transfection of pCD2.1-ciR and pCD2.1-circGHR after 48h, Edu test results. The data are represented as the means ± SEM; ** means $P < 0.01$, the difference is extremely significant, * means $P < 0.05$, significant difference.
